# Diagnostic Snapshot: "I Have a Bad Cold"

**Published:** 2018-03-01

**Authors:** Julie Kay Baker, Virginia L. Beggs

**Affiliations:** 1 Smilow Cancer Hospital at Yale New Haven;; 2 formerly of Dartmouth-Hitchcock Medical Center, Lebanon, New Hampshire

**Figure 1:**
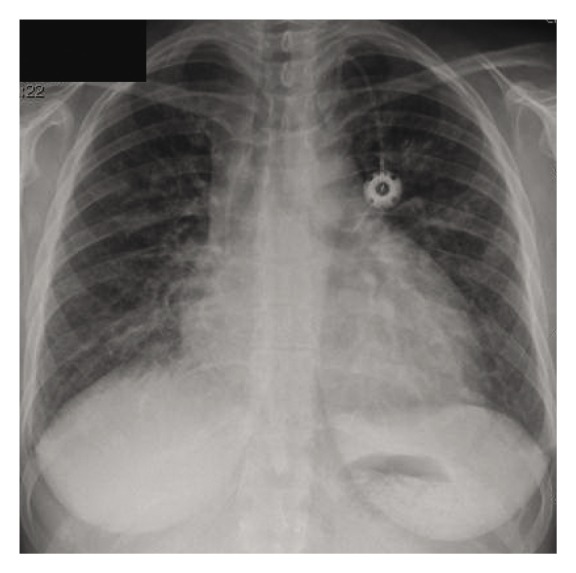
Figure 1.

Ms. J is a 37-year-old woman with a history of Hodgkin lymphoma, presenting with symptoms due to mediastinal adenopathy, diagnosed at age 12. She was treated with vincristine, prednisone, procarbazine, and doxorubicin, then 21 Gy radiation to the mediastinum. At 36, she was diagnosed with locally advanced HER2-positive breast cancer and was treated with 4 cycles of doxorubicin (total 240 mg/m² ) and cyclophosphamide, and is now receiving paclitaxel and trastuzumab; she has completed 3 courses of treatment. Her past medical history was otherwise notable for hyperlipidemia.

Ms. J presents to the oncology clinic for treatment but complains of a new-onset cough with shortness of breath and fatigue over several days. Family contacts have had similar symptoms of cough and fever, so she feels she has a bad cold.

## EXAMINATION AND WORKUP

On exam, Ms. J appears fatigued. She is mildly dyspneic when ambulating over and up onto exam table. There is postpharyngeal erythema and no exudates. There is no cervical adenopathy. Her chest has decreased breath sounds in the bases and scattered rhonchi. Her abdomen is soft, with her liver palpable on deep inspiration. Her heart S1, S2 rate is 120 bpm, and her extremities are with 1+ edema.

Ms. J’s vital signs are temperature 38.8 °C, blood pressure 110/80 mm Hg, respirations 24/min and slightly labored with exertion, O2 saturation level 93%, weight is 145 lb, which is 5 lb above baseline.

Further workup includes a CXR showing mild cardiomegaly with small pleural effusions bilaterally. ECG shows sinus tachycardia with narrow QRS and no other changes. Nasopharyngeal DFA swab is positive for influenza A. Her white count is 9,000/mm³ with an elevated lymphocyte count. Pro–B-type natriuretic peptide (BNP) is elevated at 15,000 pg/mL. A TTE performed later that afternoon shows mildly increased left ventricular size and moderately decreased systolic function due to moderate global hypokinesis. Estimated LVEF is 39%. There is mild mitral regurgitation. Right ventricular pressure is normal at 23 mm Hg. Compared to her ECG 3 months prior, her LVEF has dropped from 51%.

**Figure 2:**
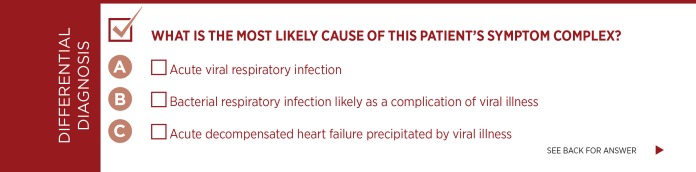
What is the most likely cause of this patient’s symptom complex?

## CORRECT ANSWER: C

**Acute decompensated heart failure precipitated by viral illness.** Ms. J’s complaints of fatigue and cough could be attributed to acute viral illness, but could also be signs of heart failure (HF). Dyspnea is due to volume overload due to congestive HF. Other signs of volume overload include lower extremity edema and weight gain, with palpable liver related to elevated jugular venous pressure. Signs of more significant cardiomegaly include auscultation of S3 and apical impulse displacement lateral to the midclavicular line and elevated jugular venous pulse. Decreased breath sounds were due to pleural effusions in the bases, but presentation could have otherwise been noted for rales should there have been an elevated right heart filling pressure.

Chest radiograph endorsed HF evidenced by cardiomegaly and effusions, and CXR could have shown pulmonary congestion due to pulmonary venous hypertension, as in Figure 1, which also depicts cardiomegaly. Electrocardiogram evidenced tachycardia that could in part be related to acute infection and fever but also as compensation of HF. A narrow QRS complex can be indicative of heart strain when seen with other ECG abnormalities such as ST changes. The cardiac biomarker proBNP reflects cardiac stress and is often elevated in HF. While she has influenza, presentation is highly suggestive of underlying congestive HF with acute decompensation due to her illness. The TTE showed definitive evidence of acute HF with a drop in LVEF from normal baseline to 39%. Superimposed bacterial infection is a possibility but does not explain symptoms of HF, nor is supported by clinical signs (lack of exudates, adenopathy, consolidations on CXR, with lymphocytosis and no neutrophilia). Influenza virus rarely causes cardiomyopathy, and bacterial infections (by blood culture) should be ruled out as a source of cardiomyopathy.

Ms. J’s history of anthracycline exposure, mediastinal radiation therapy (RT), current trastuzumab therapy, and hyperlipidemia increase the risk of heart disease. Doxorubicin, an anthracycline, is a crucial component of therapy in Hodgkin lymphoma and breast cancer. However, it is a known cardiotoxin with many proposed mechanisms of incurring cardiac damage. One often-proposed means is that of oxidative stress, leading to extracellular matrix remodeling (Nikitovic et al., 2014) through impact on mitogen active and stress active protein kinases, which are instrumental in the regulation of gene expression, survival pathways, proliferation, and growth (Geisberg & Sawyer, 2010), and so can be cardiotoxic in cumulative doses. Geisberg and Douglas (2010) describe the additional impact from anthracycline exposure causing cardiotoxicity, including the loss of endothelial cells and progenitor cells in addition to myocytes, the mechanism of sarcomere maintenance disruption and the sarcomere’s protein stability, and effects on progenitor cells in the heart.

Ms. J received treatment well under the dose limits adhered to by most clinicians of 400 to 450 mg/m²; however, having received an anthracycline in addition to RT and trastuzumab therapy increases her risk of cardiotoxicity (Rahman, Yusuf, & Ewer, 2007), and it is possible for doxorubicin given under the dose limits to incur cardiomyopathy. Radiotherapy may contribute to cardiac disease, frequently by way of valvular defects as well as coronary artery disease, myocardial disease, and conduction disorders (Schellong et al., 2010). Trastuzumab, a monoclonal antibody targeted against human epidermal growth factor and important in the treatment of HER2-positive cancers, has been associated with up to 34% cardiotoxicity, but is not dose dependent and can be reversible (Cardinale et al., 2010). It is proposed that trastuzumab-mediated inhibition may alter mitochondrial integrity that causes adenosine triphosphate depletion and contractile dysfunction, leading to cardiomyopathy (Sengupta, Northfelt, Gentile, Zamorano, & Khandheria, 2008).

## EXPLANATION OF INCORRECT ANSWERS

**Acute viral respiratory infection.** Although Ms. J does have an acute respiratory viral infection that needs to be treated, the manifestations of HF are the cause of most of her symptoms and findings. And although her prior oncologic therapies have likely contributed to the condition of HF, the acute failure she is experiencing has been triggered by infection. In comparison with other precipitating morbidities such as atrial fibrillation, HF precipitated by infection is associated with a worse outcome (Arrigo et al., 2017), and thus needs to be managed quickly and aggressively.

**Bacterial respiratory infection likely as a complication of viral illness.** Although bacterial infections can be a complication of viral infections, and although bacterial infections can precipitate HF, Ms. J’s presentation does not suggest an active bacterial infection (as described earlier in the text). Given active chemotherapy treatment, blood cultures should be obtained to rule out systemic bacterial infection, and she should be monitored carefully for the development of a bacterial pneumonia. Heart failure is the cause of her most concerning symptoms.

## TREATMENT PLAN

Ms. J’s treatment would include oseltamivir for influenza A and coordination of care with cardio-oncology. Further workup could include a cardiac magnetic resonance imaging, and possibly heart catheterization dependent upon her course (Yeh, 2016). Beta blockers and angiotensin-converting enzyme inhibitors would be part of her cardiac treatment regimen and diuretics, particularly in the short term, to reduce volume load (Yancy et al., 2013; Yeh, 2016). Trastuzumab therapy would be held and a plan for alternative care considered; however, because trastuzumab does not appear to cause significant and persistent changes in the cardiomyocytes, its effects can be reversible (Sengupta et al., 2008). It is possible that when Ms. J recovers from influenza and has a break from therapy, should TTE show recovery of ventricular function, she could be rechallenged with trastuzumab without recurrent cardiotoxicity (Sengupta et al., 2008).

## References

[1] Arrigo Mattia, Gayat Etienne, Parenica Jiri, Ishihara Shiro, Zhang Jian, Choi Dong-Ju, Park Jin Joo, Alhabib Khalid F, Sato Naoki, Miro Oscar, Maggioni Aldo P, Zhang Yuhui, Spinar Jindrich, Cohen-Solal Alain, Iwashyna Theodore J, Mebazaa Alexandre (2017). Precipitating factors and 90-day outcome of acute heart failure: a report from the intercontinental GREAT registry.. *European journal of heart failure*.

[2] Cardinale Daniela, Colombo Alessandro, Torrisi Rosalba, Sandri Maria T, Civelli Maurizio, Salvatici Michela, Lamantia Giuseppina, Colombo Nicola, Cortinovis Sarah, Dessanai Maria A, Nolè Franco, Veglia Fabrizio, Cipolla Carlo M (2010). Trastuzumab-induced cardiotoxicity: clinical and prognostic implications of troponin I evaluation.. *Journal of clinical oncology : official journal of the American Society of Clinical Oncology*.

[3] Geisberg Carrie Anna, Sawyer Douglas B (2010). Mechanisms of anthracycline cardiotoxicity and strategies to decrease cardiac damage.. *Current hypertension reports*.

[4] Nikitovic Dragana, Juranek Ivo, Wilks Martin F, Tzardi Maria, Tsatsakis Aristidis, Tzanakakis George N (2014). Anthracycline-dependent cardiotoxicity and extracellular matrix remodeling.. *Chest*.

[5] Rahman Atiar M, Yusuf Syed Wamique, Ewer Michael S (2007). Anthracycline-induced cardiotoxicity and the cardiac-sparing effect of liposomal formulation.. *International journal of nanomedicine*.

[6] Schellong Günther, Riepenhausen Marianne, Bruch Christian, Kotthoff Stefan, Vogt Johannes, Bölling Tobias, Dieckmann Karin, Pötter Richard, Heinecke Achim, Brämswig Jürgen, Dörffel Wolfgang (2010). Late valvular and other cardiac diseases after different doses of mediastinal radiotherapy for Hodgkin disease in children and adolescents: report from the longitudinal GPOH follow-up project of the German-Austrian DAL-HD studies.. *Pediatric blood & cancer*.

[7] Sengupta Partho P, Northfelt Donald W, Gentile Federico, Zamorano Jose L, Khandheria Bijoy K (2008). Trastuzumab-induced cardiotoxicity: heart failure at the crossroads.. *Mayo Clinic proceedings*.

[8] Yancy Clyde W, Jessup Mariell, Bozkurt Biykem, Butler Javed, Casey Donald E, Drazner Mark H, Fonarow Gregg C, Geraci Stephen A, Horwich Tamara, Januzzi James L, Johnson Maryl R, Kasper Edward K, Levy Wayne C, Masoudi Frederick A, McBride Patrick E, McMurray John J V, Mitchell Judith E, Peterson Pamela N, Riegel Barbara, Sam Flora, Stevenson Lynne W, Tang W H Wilson, Tsai Emily J, Wilkoff Bruce L (2013). 2013 ACCF/AHA guideline for the management of heart failure: a report of the American College of Cardiology Foundation/American Heart Association Task Force on Practice Guidelines.. *Journal of the American College of Cardiology*.

[9] Yeh E (2016). *MD Anderson practices in onco-cardiology. *.

